# Human visceral leishmaniasis and polymorphisms in interleukin-coding genes: a systematic review

**DOI:** 10.1590/1678-9199-JVATITD-2024-0018

**Published:** 2024-10-18

**Authors:** Amanda Virginia Batista Vieira, Manuela Rocha de Menezes, Pablo Cantalice Santos Farias, Elis Dionísio da Silva, Gilberto Silva Nunes Bezerra, Walter Lins Barbosa, Zulma Maria de Medeiros

**Affiliations:** 1 Graduate Program in Health Sciences, University of Pernambuco, Recife, PE, Brazil.; 2 Department of Parasitology, Aggeu Magalhães Institute, Oswaldo Cruz Foundation, Recife, PE, Brazil.; 3 Department of Genetics, Federal University of Pernambuco, Recife, PE, Brazil.; 4 Institute of Health and Biotechnology, Federal University of Amazonas , Coari, AM, Brazil.; 5 Department of Nursing & Healthcare, Technological University of the Shannon: Midlands Midwest, Athlone, Ireland.

**Keywords:** Kala-azar, Visceral leishmaniasis, *Leishmania* sp., Single base polymorphism, Systematic review

## Abstract

Visceral leishmaniasis (VL) is a neglected disease that is typical of tropical and subtropical parts of the world and is caused by the trypanosomatid *Leishmania donovani* complex. This disease is a multifactorial condition that involves parasitic, environmental, and immunogenetic characteristics. Genetic changes in genes encoding cytokines may be associated with changes in their expression and, consequently, with the development of clinical resistance or susceptibility to the disease. This systematic review and meta-analysis aimed to assess whether single nucleotide polymorphisms (SNPs) in interleukin genes influence the clinical consequences of visceral leishmaniasis infection. To this end, we carried out a systematic review and meta-analysis with structured searches in the EMBASE, PubMed, Scopus, SciELO, and Web of Science databases without time restrictions. Two independent reviewers examined the studies, performed data extraction, and assessed quality by assigning scores. If there were any discrepancies, a third reviewer with more experience was consulted. After the screening process, 28 articles were included in the systematic review and 9 in the final analysis of the meta-analysis. Statistical analyses were carried out using various genetic models. The odds ratio (OR) and corresponding 95% confidence intervals (CIs) were calculated to estimate the associations. Overall, the main clinical outcomes were classified as not associated or associated when they presented susceptibility, resistance, risk, or protective factors for the development of the disease. Associations between IFN-γ +874T/A polymorphisms in the dominant model (OR 1.64, 95% CI 1.13-2.38, I^2^ = 0%, p < 0.01) and heterozygous model (OR 1.72, 95% CI 1.15-2.57, I^2^ = 0%, p < 0.01) and IL-18 -137G/C in the recessive model (OR 1.33, 95% CI 1.02-1.71, I^2^ = 9%, p = 0.03) and VL were observed. For the IL-10 gene SNPs, there was no significant association. Our findings suggest that SNPs in the IFN-γ and IL-18 genes may be associated with the risk of developing VL.

## Background

Visceral leishmaniasis (VL) or kala-azar is a neglected disease typical of tropical and subtropical regions of the world. This disease is caused by trypanosomatid protozoa of the *Leishmania donovani* complex and can be fatal when early treatment is not administered [ 1- 2]. It is a vector-borne disease transmitted by sandflies of the genera *Lutzomyia* and *Phlebotomus* from the New and Old Worlds, respectively. According to the World Health Organization (WHO), there are 50 to 90,000 new cases annually; however, most of them are underreported, and only 25 to 45% of them are informed to the world health authority. More than 90% of these are concentrated in 10 countries: Brazil, China, Ethiopia, Eritrea, India, Kenya, Somalia, South Sudan, Sudan, and Yemen [ 2].

The development of the disease is induced by several factors, such as the environment, the pathogen, and host factors. Regarding the hosts, genetic and immune characteristics may trigger resistance or susceptibility to VL. Host genetic variations may be classified as changes in structure, copy number, transposon, insertion/deletion, or single nucleotide polymorphism (SNP) [ 3]. SNPs are single nucleotide changes that are present in more than 1% of the world's population. These factors may change the functions of promoter regions, inducing an increase or decrease in the production of gene transcripts or modifying the proteins themselves [ 3, 4].

Furthermore, the presence of polymorphisms in genes that encode interleukins may directly affect the type of immune response, interfere with their production levels, and, consequently, susceptibility or resistance to the disease [ 3- 5]. The intracellular location of the parasites triggers an immune response mediated by CD4+ lymphocytes; however, there is a duality, in which conventionally the Th1 profile is associated with resistance to the disease, with the presence of cytokines such as IFN-γ, IL-12, IL-2. The Th2 response is associated with susceptibility and with the synthesis of interleukins such as IL-4, IL-5, IL-10, and IL-13. The high production of Th1 cytokines may contribute to an exacerbated inflammatory response and to the pathogenesis of the disease. Therefore, this response is normally accompanied by regulatory T cells (Tregs), which synthesize IL-10 and TGF-β, for example, and act to modulate the immune response and prevent tissue damage. In addition, Th17 cells differentiate in the presence of TGF-β and IL-6 and synthesize interleukins such as IL-17 and IL-22, but their roles in the pathogenesis of this disease are still contradictory [ 6].

Therefore, the presence of SNPs in genes of interleukins may alter production levels and, consequently, the conditions for the development of the disease [ 3, 4, 5]. Thus, this study aimed to assess whether SNPs in interleukin genes influence the clinical consequences of visceral leishmaniasis by a systematic review and meta-analysis of all eligible studies, which may yield more accurate and robust estimates of the association between the polymorphisms and clinical outcome.

## Methods

### Registry protocol

In this systematic review, we adhered to the PRISMA 2020 guidelines and checklist -Preferred Reporting Items for Systematic Reviews and Meta-Analyses [ 7] to reduce the possibility of insertion of biases. Our methodology protocol was registered in the Prospective International Registry Platform for Systematic Reviews (PROSPERO/National Institute for Health Research - CRD42022350889) (Additional file 1).

### Data sources and search strategy

A bibliographic survey of scientific articles was carried out between March and April 2024 without restriction on publication date. The databases used in the searches were EMBASE (Elsevier), PubMed (National Center for Biotechnology Information), Scopus (Elsevier), SciELO, and Web of Science (Clarivate). The search key and its MeSH terms were as follows: (“visceral leishmaniasis” OR “black fever” OR “fever-black” OR “kala-azar” OR “kala azar” OR “leishmaniasis, visceral” [Mesh]) AND (“gene polymorphism” OR “gene polymorphisms” OR “polymorphism, gene” OR “polymorphisms, gene” OR “genetic polymorphism” OR “genetic polymorphisms” OR “polymorphism (genetic)” OR “polymorphisms (genetics)” OR “polymorphisms, genetic” OR “polymorphism, genetic” [MESH]) AND (human OR “humans” [Mesh]) (Additional file 2). Hand-searching for studies was also carried out to identify studies not captured by the search key. The complete methodology with more details is available on the PROSPERO website (https://www.crd.york.ac.uk/prospero/display_record.php? ID=CRD42022350889) [ 8].

### Eligibility criteria

Studies were considered eligible if they involved SNPs in genes encoding cytokines in individuals with VL. Unqualified papers were excluded if they met the following criteria: (i) research carried out with non-human VL; (ii) studies carried out with other types of leishmaniasis other than its visceral form; (iii) articles that focused only on bioinformatics analyses; and (iv) narrative reviews, systematic (with or without meta-analysis), editorials, conference abstracts, case reports, or books.

### Study selection and data extraction

The references were imported into Rayyan online software [ 9] for data management. Two independent reviewers (AVBV and MRM) were responsible for the screening process. Initially, duplicates were removed. The first phase of selection was based on the analysis of titles and abstracts according to eligibility criteria. Subsequently, the second phase of the selection was based on the reading of the full texts of potentially eligible studies and reanalysis of the inclusion criteria. In the presence of any disagreements, a more experienced third reviewer (ZMM) assessed the data. Considering the eligibility criteria, the following data were extracted: (i) name(s) of the author(s), (ii) year of publication, (iii) polymorphism, (iv) SNP location, (v) genotyping method, and (vi) outcome. This extraction was performed independently by two reviewers (AVBV and MRM). In the presence of any discrepancies, a third reviewer (ZMM) checked the data.

### Risk of bias and quality assessment

The selected studies were independently analyzed by two reviewers (AVBV and MRM) using the Standard Quality Assessment Criteria for Evaluation of Primary Research Papers from a Variety of Fields [ 10], a tool for systematically evaluating reviews. The protocol is composed of quantitative analysis, where 14 assessment items are used. The scores range from 0 to 2 points, in which “0” represents no, “1” represents partial, and “2” represents yes. The questions answered by N/A (not applicable) were not part of the calculation of the total points score (number of “yes” * 2 + number of “partials” * 1) or maximum (28 - number of “N/A” * 2). The quality of the studies was analyzed from the value of the final percentage; the higher this number was, the lower the risk of bias. In the presence of any disagreements, a third reviewer (ZMM) evaluated the studies.

### Statistical analysis

The associations between polymorphisms and the development of VL were evaluated using comparative genetic models: dominant, recessive, allelic, homozygous, and heterozygous. The odds ratio (OR) values were used to test the association between each SNP and disease association in the studies. The 95% confidence intervals and p values < 0.05 were considered to indicate statistical significance. Cochran’s Q statistic and the I^2^ test were used to evaluate heterogeneity [ 11]. In the presence of high heterogeneity values (I^2^ > 50%), the random effects model was used; otherwise, the fixed effects model was applied [ 12]. The chi-square test was applied to examine the Hardy‒Weinberg equilibrium (HWE) in the healthy control groups in the different studies with PLINK v1.9 software. HWE was considered when p > 0.05. All tests in this meta-analysis were performed with RStudio software version 4.4.0 [ 13].

## Results

### Flow of included studies

**Figure 1.**
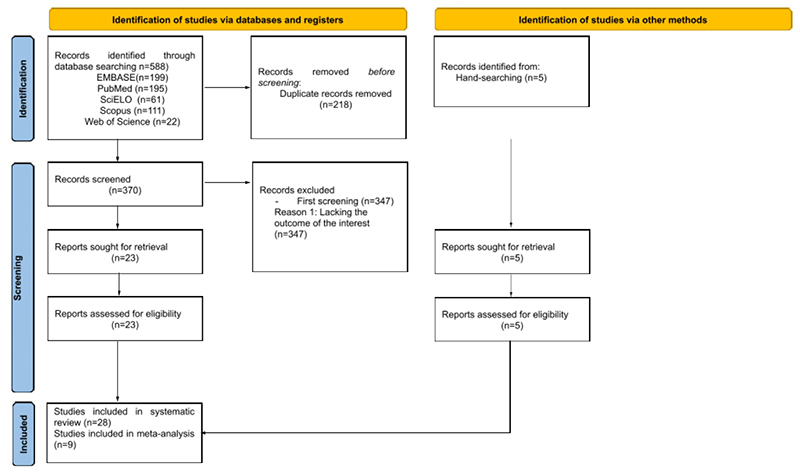
Preferred Reporting Items for Systematic Reviews and Meta-Analyses (PRISMA) flow diagram displaying the systematic search and review process.

The study selection process is summarized in the flow diagram of the Preferred Reporting Items for Systematic Reviews and Meta-Analysis (PRISMA) shown in Figure 1. The initial search yielded 588 articles (199 in EMBASE, 195 in PubMed, 61 in SciELO, 111 in Scopus, and 22 in Web of Science). Of these, 218 were excluded because they were duplicated. After this exclusion, 370 articles remained and passed to the first screening (reading of titles and abstracts), in which the inclusion criteria were verified and 347 were excluded for not following the objective of the study. Subsequently, full texts of the 23 previously selected studies were read, all of which were included because they fully met the inclusion criteria. Manual searches were also carried out in the reference lists of previously selected articles, with 5 studies included using this methodology. At the end of the entire selection process, 28 articles were included in this systematic review, 9 of which were included in the meta-analysis stage.

### Description of the studies

The characteristics of all included articles are shown in Table 1. The selected articles were published from 2002 to 2021. Case-control studies, with comparison groups (VL carriers, asymptomatic and healthy) and using family members were carried out in 14, 11, and 3 articles, respectively. Sample sizes ranged from small groups of 15 cases to studies with more than 1000 individuals divided between families. The study areas of the inserted articles were Brazil = 3, India = 4, Iran = 15, Iraq = 3, Morocco = 1, Mexico = 1 and Sudan = 1.

**Table 1.** Details of studies that evaluated polymorphisms in VL-associated cytokine genes.

References	Country	Species of protozoan	Genotyping method	SNP location	Outcome	Polymorphism
**Moravej*et al.*** [14]	Iran	*Leishmania infantum*	PCR-RFLP	Exon	Associated [Susceptibility (-511CC genotype and CC haplotype (-511/+3953) and resistance (-511 TT genotype) and tde TT haplotype (-511/+3953)]	*+3953 (IL-1β)*
				Promoter		*− 511 (IL-1β)*
**Ahmadi*et al*.** [15]	Iran	*Leishmania infantum*	PCR-RFLP	3'UTR	Associated (risk)	*rs1126647 (IL-8)*
**Hajilooi*et al.*** [16]	Iran	*Leishmania infantum*	AS-PCR	Promoter	Associated	*rs4073 (IL-8)*
**Frade*et al*.** [17]	Brazil	*Leishmania infantum*	PCR-RFLP	Promoter	Not associated	*rs4073 (IL-8)*
**Frade*et al.*** [17]	Brazil	*Leishmania infantum*	PCR-RFLP	Promoter	Associated [susceptibility (-509 T allele)]	*rs1800469 (TGFB1)*
				Exon		*rs1800470 (TGFB1)*
**Kalani*et al*.** [18]	Iran	*Leishmania infantum*	PCR-RFLP	3' UTR	Not associated	*+1188 (IL-12)*
**Kalani*et al*.** [18]	Iran	*Leishmania infantum*	AS-PCR	Intron	Associated [susceptibility (AT genotype) and resistance (TT resistance)]	*rs2430561 (IFN-γ)*
**Kalani*et al.*** [18]	Iran	*Leishmania infantum*	PCR-RFLP	Promoter	Not associated	rs1800872 (*IL-10*)
						*rs1800871 IL-10*)
						rs1800896 (*IL-10*)
**Zahra’a *et al*.** [19]	Iraq	*Leishmania infantum*	Cytokine CTS-PCR-SSP Tray Kit	Promoter	Not associated	*rs3212227 (IL-12B)*
**Rasouli *et al*.** [20]	Iran	*Leishmania infantum*	PCR-RFLP	Exon	Associated (Risk)	13.687 (*IL-15)*
				Intron	Not associated	367 (*IL-15)*
					Associated (Risk)	267 (*IL-15)*
				3' UTR	Not associated	14,035 (*IL-15)*
**Kumar *et al*.** [21]	India	*Leishmania donovani*	PCR-RFLP	Promoter	Associated [protective (G allele)]	*rs1946519 (IL-18)*
					Not associated	rs187238*(IL-18)*
				Codon region	Not associated	*rs549908 (IL-18)*
Moravej et al [22]	Iran	*Leishmania infantum*	PCR-RFLP	Promoter	Not associated	rs187238*(IL-18)*
				Codon region		*rs549908 (IL-18)*
**Ahmadpour *et al.*** [23]	Iran	*Leishmania infantum*	ARMS-PCR	Promoter	Associated [resistance (CC genotype)]	*rs1946518 (IL-18)*
					Not associated	rs187238*(IL-18)*
**Babaloo *et al.*** [24]	Iran	*Leishmania infantum*	ARMS-PCR	Promoter	Not associated	*rs2227473 (IL-22)*
**Kalani *et al*.** [25]	Iran	*Leishmania infantum*	PCR-RFLP	Intron	Not associated	*rs2227501 (IL-22)*
				Intron	Associated (susceptibility AG genotype)	*rs2227513 (IL-22)*
				3' UTR	Associated [allele A and the AA can be considered as a protective factor against the development]	*rs1026786 (IL-22)*
**Karplus *et al*.** [26]	Brazil	*Leishmania infantum*	PCR-RFLP	Promoter	Associated	*-307 (TNF)*
**Ejghal *et al.*** [27]	Morocco	*Leishmania infantum*	PCR-RFLP	Promoter	Not associated	rs1800629 (*TNF-α)*
				Intron	Not associated	*rs361525 (TNF-β)*
**Ortiz-Flores *et al*.** [28]	Mexico	*Leishmania infantum*	PCR-RFLP	Promoter	Not associated	-308*(TNF)*
					Not associated	-238*(TNF)*
**Maghsood *et al*.** [29]	Iran	*Leishmania infantum*	ARMS-PCR	Intron	Not associated	*rs2430561 (IFN-γ)*
Al-Bashier [30]	Iraq	*Leishmania infantum*	ARMS-PCR	Intron	Associated [risk factor (allele A)]	*rs2430561 (IFN-γ)*
Al-Bashier [30]	Iraq	*Leishmania infantum*	ARMS-PCR	Promoter	Not associated	rs1800896 (*IL-10)*
**Mishra *et al*.** [31]	India	*Leishmania donovani*	PCR-Sequencing	Promoter	Not associated	rs2243250*(IL-4)*
						rs2070874*(IL-4)*
				Intron		rs79071878 (IL-4)
**Mohamed *et al.*** [32]	Sudan	*Leishmania donovani*	PCR-Sequencing	Intron	Associated	IL4RP2
						IL4RP1
**Jerônimo *et al*.** [33]	Brazil	*Leishmania infantum*	TaqMan SNP genotyping	Promoter	Associated	*rs2070874 (IL-4)*
**Hajilooi *et al.*** [34]	Iran	*Leishmania infantum*	PCR-RFLP	Promoter	Associated [Risk factor (C/T)]	*rs1800871 (IL-10)*
**Ahmed *et al*.** [35]	Iraq	*Leishmania infantum*	Cytokine CTS-PCR-SSP Tray Kit	Promoter	Not associated	*rs1800896 (IL-10)*
						*rs1800871 (IL-10*
						*rs1800872 (IL-10)*
**Hajilooi *et al.*** [36]	Iran	*Leishmania infantum*	PCR-RFLP	Promoter	Associated 472. [Risk factor (A/G)]	rs1800896 (*IL-10)*
**Mishra *et al*.** [37]	India	*Leishmania donovani*	PCR-Sequencing	Intron	Associated (risk-reducing G allele and the AG and GG genotypes related to protection)	*rs1518111(IL-10)*
				Intron		*rs1554286 (IL-10)*
				3'UTR		*rs3024496 (IL-10)*
				3'UTR		*rs3024498 (IL-10)*
**Hamidi *et al.*** [38]	Iran	*Leishmania infantum*	ARMS-PCR	Promoter	Associated [susceptibility (-509 T allele)]	*rs1800469 (TGFB1)*
**Rasouli *et al*.** [39]	Iran	*Leishmania infantum*	PCR-RFLP	Promoter	Associated (resistance)	*rs3819024 (IL-17-A)*
				Intron	Associated (resistance)	*rs3819025 (IL-17A)*
				Intron	Associated (resistance)	*rs8193038 (IL-17A)*
				3’UTR	Associated [susceptibility (AA genotype and A allele)]	*rs3748067 (IL-17A)*
**Khatonier *et al*.** [40]	India	*Leishmania donovani*	PCR-RFLP	Promoter	Associated [susceptibility (rs8193036TT genotype and the T allele)]	*rs2275913 (IL-17A)*
						*rs8193036 (IL-17A)*
**Sadighi *et al.*** [41]	Iran	*Leishmania infantum*	ARMS-PCR	3’UTR	Not associated	*rs1974226 (IL-17A)*

AS-PCR: allele-specific polymerase chain reaction, ARMS-PCR: amplification refractory mutation system-polymerase chain reaction, PCR-RFLP: polymerase chain reaction-restriction fragment length polymorphism.

The methods used for genotyping the individuals in each study varied and included the following: AS-PCR: allele-specific polymerase chain reaction (2); ARMS-PCR: amplification refractory mutation system-polymerase chain reaction (7); cytokine CTS-PCR-SSP Tray Kit (2); PCR-Sequencing (3); PCR-RFLP: polymerase chain reaction-restriction fragment length polymorphism (17); and TaqMan SNP genotyping (1).

The SNPs studied are from different gene regions, such as 3 exons, 13 introns, 22 promoters, 8 3'UTRs, and 2 codon regions. For 22 SNPs, there was no association with the development of VL. In 24 of them, there was the presence of an outcome of disease (susceptibility, resistance, and risk or protective factor). Most studies (23) were carried out where the species *L. infantum* is endemic. In the other studies (5), the parasite responsible for infection was *L. donovani*.

### SNPs associated with clinical outcomes of the disease

- **511T/C and +3953 T/C polymorphisms**

Moravej *et al.* [ 14] concluded that the -511CC genotype and CC haplotype (-511/+3953) of the *IL-1β* gene were related to susceptibility to the development of the disease. In contrast, the -511 TT genotype and the TT haplotype (-511/+3953) were associated with resistance factors in the Iranian population. Furthermore, the serum levels of this interleukin were greater in individuals with the CC genotype than in those with the TT genotype.

rs1126647 and rs4073 polymorphisms

Ahmadi *et al.* [ 15], when studying Iranian individuals, concluded that genotype +2767 A/A of the IL-8 gene is associated with positive regulation of this cytokine in patients with VL. Furthermore, Hajilooi *et al.* [ 16], when evaluating the same population, concluded that the TT genotype in the -251 T/A region is considered a risk factor for the development of VL. In Brazilians, there was no association between this SNP and the disease [ 17].

+1188 and -1188 polymorphisms

In a study carried out by Kalani *et al.* [ 18] using the +1188 region of the IL-12 gene, there was no significant association between polymorphisms and disease development in an Iranian population. However, Zahra'a *et al.* [ 19], when evaluating the -1188 C/A of IL-12B, concluded that although this SNP was not associated with resistance or susceptibility, the serum levels of this interleukin were greater in patients with the CC genotype than in those with the other genotypes.

267 and 13687 polymorphisms

Rasouli *et al.* [ 20] concluded that SNPs at positions 267 and 13687 of the IL-15 gene were associated with the risk of visceral leishmaniasis in Iranians.

rs1946519, rs1946518, rs187238 polymorphisms

Kumar *et al*. [ 21], when evaluating rs1946519 (-656) of the IL-18 gene, concluded that the G allele may be associated with protection against VL in the Indian population; however, Moravej *et al.* [ 22], when evaluating the same region, concluded that the T allele may be a resistance factor in Iranian individuals. Furthermore, Ahmadpour *et al*. [ 23] concluded that Iranians carrying the CC genotype at position -607 have greater resistance to VL infection. Furthermore, Kumar *et al.* [ 21], Ahmadpour *et al*. [ 23], and Moravej *et al*. [ 22], when analyzing the -137G/C region of the IL-18 gene, found no association with the development of the disease.

rs2227473 and rs1026786 polymorphisms

Babaloo *et al*. [ 24], when studying the SNP rs2227473 A/G of the IL-22 gene in an Iranian population, found no significant association with the development of VL. However, Kalani *et al*. [ 25] concluded that the A allele and the AA genotype of rs1026786 may be protective factors against the disease.

-307 and rs1800629 polymorphisms

Karplus *et al*. [ 26] when evaluating the genetic variability in the TNF locus and the development of VL in a Brazilian population, concluded that there is a strong association between the -307 promoter region and asymptomatic infection. When analyzing the SNP rs1800629, Ejghal *et al.* [ 27] and Ortiz-Flores *et al.* [ 28] observed that there is no association with disease progression.

rs2430561 polymorphism

Maghsood *et al.* [ 29], when studying the +874 A/T region of IFN-γ in an Iranian population, reported that there were no significant associations between resistance and susceptibility. However, the highest frequency of the wild-type genotype T was found in healthy VL seropositive individuals. As in a previous study, Kalani *et al.* [ 18] demonstrated that carriers of the TT genotype have a greater amount of circulating IFN-γ. Thus, these individuals may be more resistant to VL than to AT and AA. In addition, the presence of the AT genotype was considered a predictive factor for the susceptibility of individuals from the endemic area of Iran. However, Al-Bashier [ 30], when evaluating the same region in Iraqi individuals, concluded that carriers of the mutant allele A had a 2.54-fold increase in the risk of developing the disease compared with those with the wild-type T allele. Moreover, the +874A allele was considered a risk factor for the development of VL.

rs2243250, rs2070874, rs79071878, IL4RP2 and IL4RP1 polymorphisms

Mishra *et al.* [ 31], when studying an Indian population, did not observe an association between the SNPs rs2243250, rs2070874, and rs79071878 and the development of the disease. However, Mohamed *et al.* [ 32], when conducting a study in Sudan, showed a positive association between the SNPs IL4RP2 and IL4RP1 and the development of VL. In a study in Brazil, Jerônimo *et al.* [ 33] noted an association between IL-4 34C/T (rs2070874) and delayed-type hypersensitivity (DTH).

rs1800871, rs1800896 and rs3024498 polymorphisms

When evaluating the SNP -819 (rs1800871) of the IL-10 gene, Hajilooi *et al*. [ 34] observed a significant association between the polymorphism and VL. The CT genotype was more common in the group of patients than in the other groups (asymptomatic and healthy). This may suggest that the presence of this genetic characteristic is a possible risk factor for the development of the disease. When evaluating the same region, Kalani *et al.* [ 18] and Ahmed *et al.* [ 35] did not find a significant association.

Hajilooi *et al*. [ 36] reported that the AG genotype of region -1082 (rs1800896), which could influence the expression of this interleukin, was also considered a risk factor for the disease. Other studies, such as Ahmed *et al.* [ 35], Al-Bashier [ 30], and Kalani *et al.* [ 18], on the same region of the IL-10 gene, did not find an association between the SNPs and the development of the disease.

Moreover, Mishra *et al.* [ 37], when studying Indian individuals, concluded that the rs3024498 polymorphism (5311A > G, 3'UTR) is associated with VL. The presence of the risk-reducing G allele and the AG and GG genotypes are related to protection against the disease.

rs1800469 polymorphism

Frade *et al.* [ 17], when analyzing the -509 CT gene region of TGF-β1 in a Brazilian population, concluded that this SNP was associated with general susceptibility to infection and severity of the clinical disease. The presence of the T allele results in a 1.9-fold greater risk of developing the disease in individuals with VL and asymptomatic ones than in individuals without the presence of infection. In addition, the presence of this allele was also significantly related to the bleeding conditions of individuals with VL in this population. Hamidi *et al.* [ 38], when evaluating the same region, obtained similar results regarding the presence of the genotype and susceptibility to the disease.

rs3819024, rs3819025, rs8193038, rs3748067, rs8193036 and rs2275913 polymorphisms

Rasouli *et al.* [ 39], when evaluating the IL-17 gene, concluded that the AA genotype of the SNPs rs3819024, rs3819025, and rs8193038 are considered resistance markers and that the A allele and AA genotype of rs3748067 are susceptibility factors for the development of VL in an Iranian population. Khatonier *et al.* [ 40] evaluated an Indian population and reported that individuals carrying the rs8193036TT genotype and the T allele are more susceptible to the disease. The rs2275913A allele is considered a susceptibility factor. Another study carried out by Sadighi *et al.* [ 41], with other SNPs and again in an Iranian population, did not obtain significant results.

### Meta-analysis results

rs2430561 polymorphism

**Figure 2.**
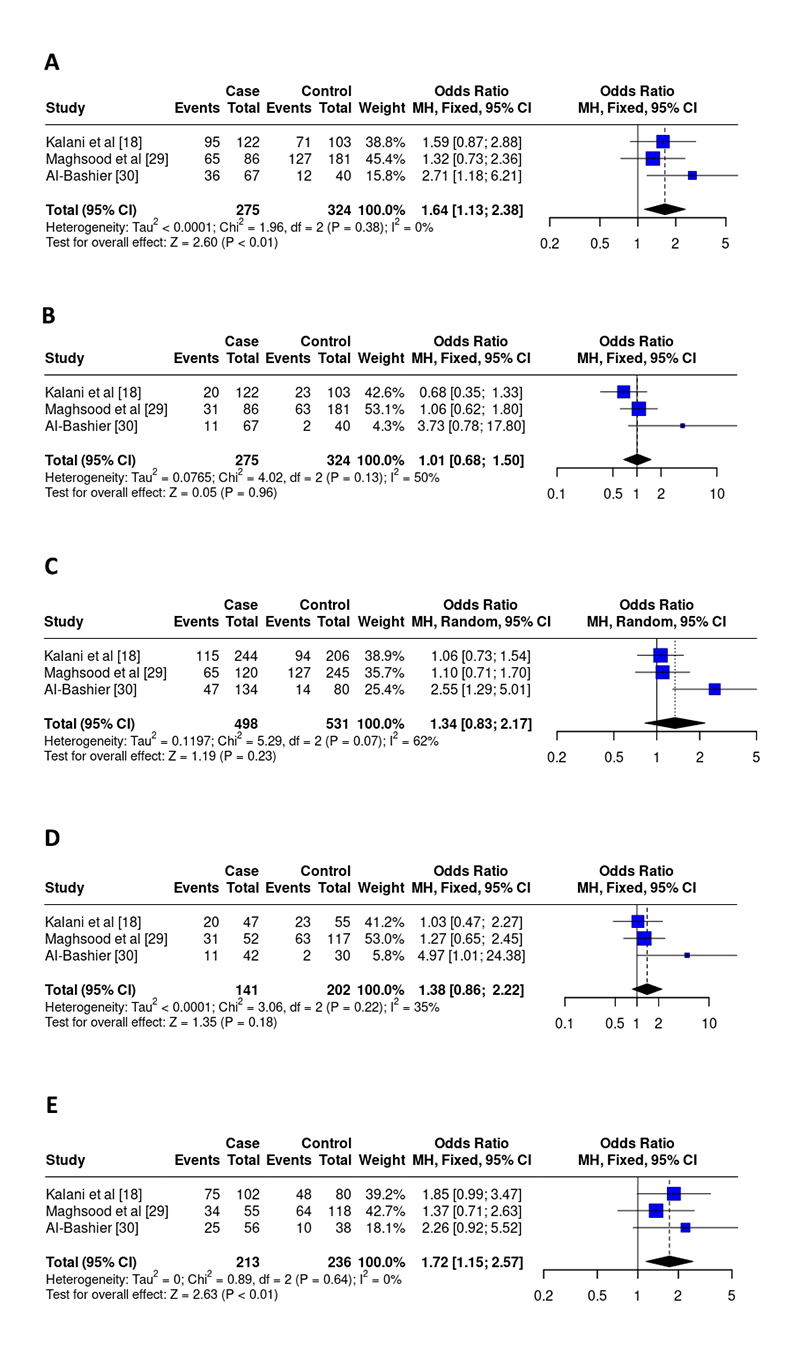
Odds ratios and 95% confidence intervals for the associations between the rs2430561 polymorphism and the progression of leishmaniasis in the models: **(A)** dominant (AA+AT vs. TT), **(B)** recessive (AA vs. TA+TT), **(C)** allelic (A vs. T), **(D)** homozygous (AA vs. TT), and **(E)** heterozygous (AT vs. TT).

For the IFN-γ SNP, 3 studies, including 275 cases and 324 controls, were counted in the final analysis [ 18, 29, 30]. There was a significant association between the dominant model (OR 1.64, 95% CI 1.13-2.38, I^2^ = 0%, p < 0.01) and the heterozygous model (OR 1.72, 95% CI 1.15-2.57, I^2^ = 0%, p < 0.01) and disease progression. For the other models, there were no associations: recessive (OR 1.01, 95% CI 0.68-1.50, I^2^ = 50%, p = 0.96), allelic (OR 1.34, 95% CI 0.83-2.17, I^2^ = 62%, p = 0.23) and, homozygous (OR 1.38, 95% CI 0.86-2.22, I^2^ = 35%, p = 0.18) ( Figure 2). Furthermore, a control group was not in the HWE ( Table 2).

**Table 2.** Distribution of genotwypes and alleles from the studies included in the meta-analysis and HWE of controls.

	Cases	Control									HWE	MAF
	AA	AT	TT	A	T	AA	AT	TT	A	T		
**Study**												
**rs2430561**												
**Kalani *et al.* 18**	20	75	27	115	129	23	48	32	94	112	0.55	0.46
**Maghsood *et al* [29]**	31	34	21	65	55	63	64	54	127	118	< 0.001	0.52
**Al-Bashier [30]**	11	25	31	47	87	2	10	28	14	66	0.32	0.18
	**Cases**	**Control**									**HWE**	**MAF**
	**GG**	**GC**	**CC**	**G**	**C**	**GG**	**GC**	**CC**	**G**	**C**		
**Study**												
**rs187238**												
**Kumar *et al.* [21]**	128	63	13	319	89	136	112	19	384	150	0.65	0.72
**Moravej *et al.* [22]**	73	36	9	182	54	93	50	13	236	76	0.13	0.76
**Ahmadpour *et al.* [23]**	59	22	10	81	32	116	52	17	169	69	0.006	0.67
	**Cases**	**Control**									**HWE**	**MAF**
	**GG**	**GA**	**AA**	**G**	**A**	**GG**	**GA**	**AA**	**G**	**A**		
**Study**												
**rs1800896**												
**Kalani *et al.* [18]**	16	44	60	76	164	19	33	50	71	113	0.004	0.35
Al-Bashier [30]	17	29	21	63	71	6	22	12	34	46	0.53	0.43
**Ahmed *et al.* [35]**	4	19	21	27	61	4	21	15	30	50	0.51	0.36
**Hajilooi *et al.* [36]**	8	102	0	118	102	35	142	10	212	162	< 0.001	0.57
	**Cases**	**Control**									**HWE**	**MAF**
	**TT**	**TC**	**CC**	**T**	**C**	**TT**	**TC**	**CC**	**T**	**C**		
**Study**												
**rs1800871**												
**Kalani *et al.* [18]**	11	45	64	67	173	11	32	59	54	150	0.07	0.26
**Hajilooi *et al.* [34]**	2	96	2	100	100	28	140	22	184	196	< 0.001	0.52
**Ahmed *et al.* [35]**	11	14	19	36	52	6	15	19	27	53	0.30	0.34

HWE: Hardy-Weinberg equilibrium; MAF: minor allele frequency.

rs187238 polymorphism

**Figure 3.**
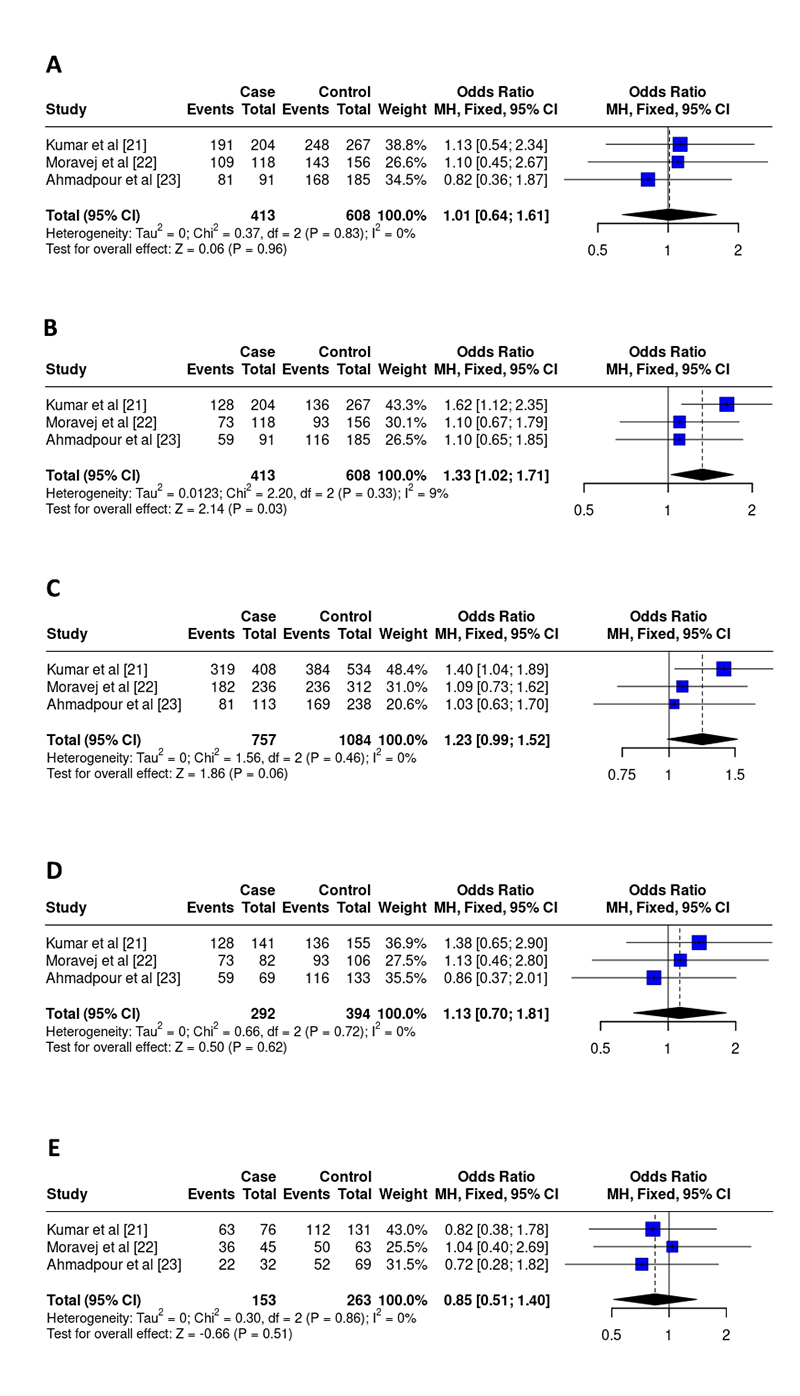
Odds ratios and 95% confidence intervals for the association between the rs187238 polymorphism and the progression of leishmaniasis in the models: **(A)** dominant (GG+GC vs. CC), **(B)** recessive (GG vs GC+CC), **(C)** allelic (G vs C), **(D)** homozygous (GG vs CC), and **(E)** heterozygous (GC vs CC).

For the IL-18 SNP, 3 studies, including 413 cases and 608 controls, were included in the final analysis [ 21, 22, 23]. There was a significant association between the recessive model (OR 1.33, 95% CI 1.02-1.71, I^2^ = 9%, p = 0.03) and disease progression. For the other models, there were no associations: dominant (OR 1.01, 95% CI 0.64-1.61, I^2^ = 0%, p = 0.96), allelic (OR 1.23, 95% CI 0.99-1.52, I^2^ = 0%, p = 0.06), homozygous (OR 1.13, 95% CI 0.70-1.81, I^2^ = 0%, p = 0.62), heterozygous (OR 0.85, 95% CI 0.51-1.40, I^2^ = 0%, p = 0.51) ( Figure 3). Additionally, a control group was not in the HWE ( Table 2).

rs1800871 polymorphism

**Figure 4.**
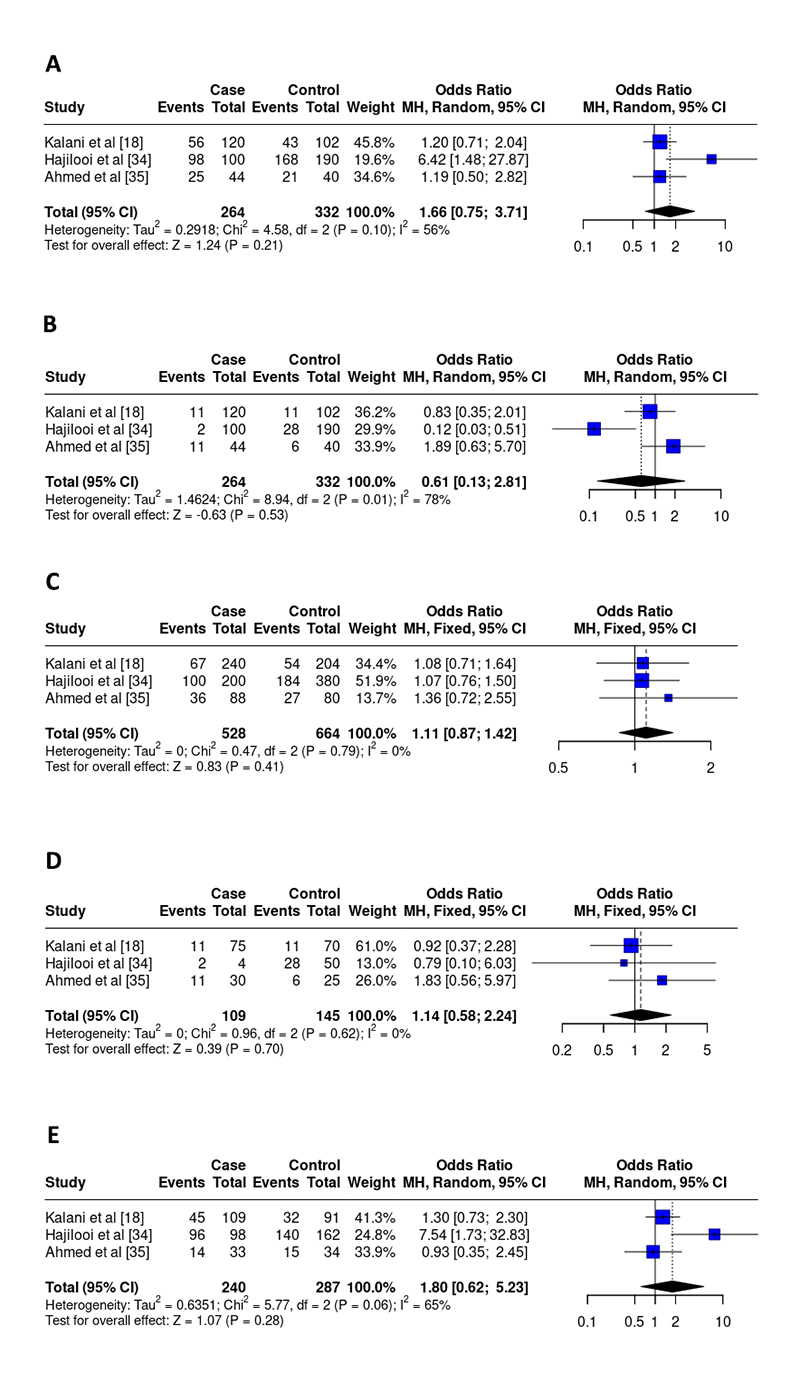
Odds ratios and 95% confidence intervals for the association between the rs1800871 polymorphism and the progression of leishmaniasis in the models: **(A)** dominant (TT+TC vs. CC), **(B)** recessive (TT vs. TC+CC), **(C)** allelic (T vs. C), **(D)** homozygous (TT vs. CC), and **(E)** heterozygous (TC vs. CC).

For the IL-10 SNP -819, 3 studies [ 18, 34, 35] encompassing 264 patients and 332 controls were included in the final analysis. No associations were detected between the development of VL and the rs1800871 SNP in any of the genetic models: dominant (OR 1.66, 95% CI 0.75-3.71, I^2^ = 56%, p = 0.21), recessive (OR 0.61, 95% CI 0.13 -2.81, I^2^ = 78%, p = 0.53), allelic (OR 1.11, 95% CI 0.87-1.42, I^2^ = 0%, p = 0.41), homozygous (OR 1.14, 95% CI 0.58-2.24, I^2^ = 0%, p = 0.70), heterozygous (OR 1.80, 95% CI 0.62-5.23, I^2^ = 65%, p = 0.28) ( Figure 4). Furthermore, a control group was not in HWE ( Table 2).

rs1800896 polymorphism

**Figure 5.**
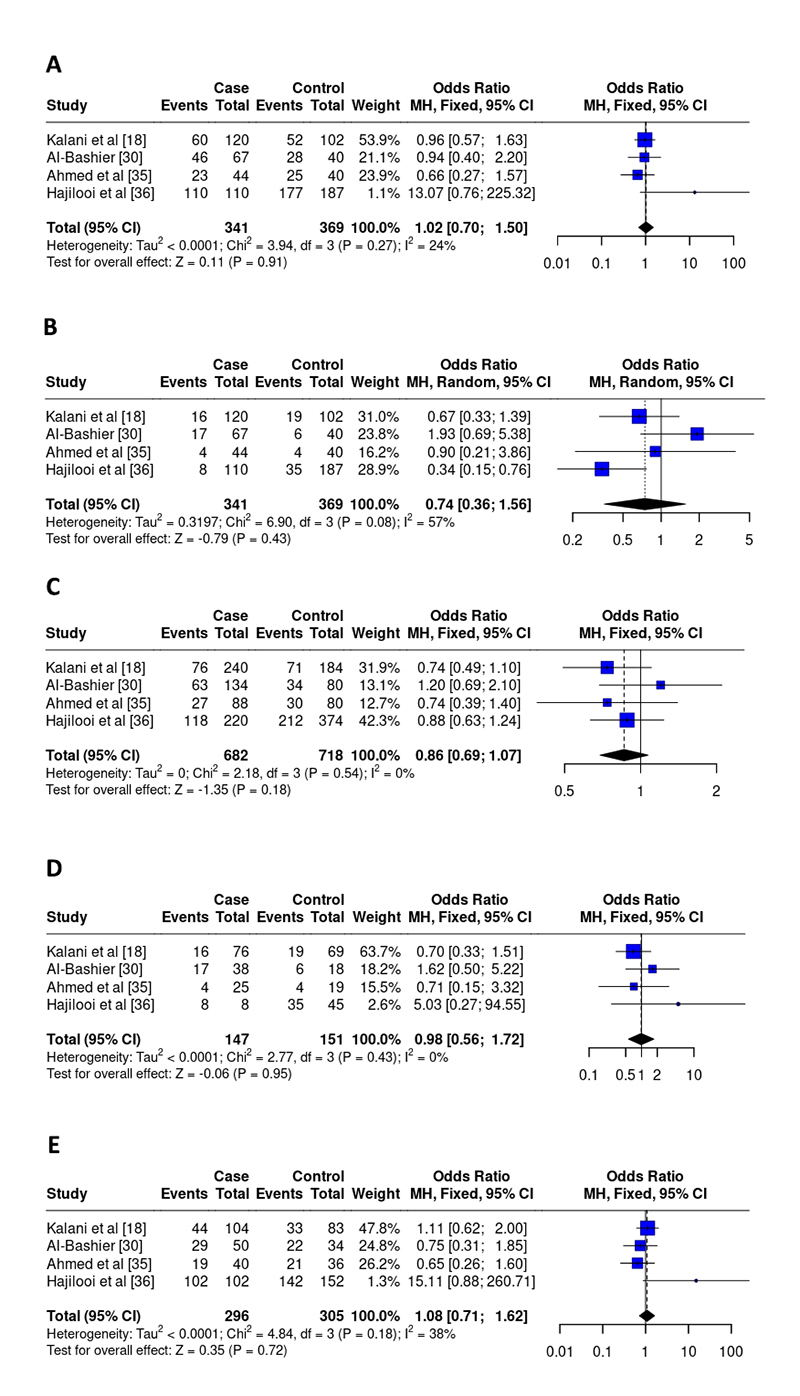
Odds ratios and 95% confidence intervals for the association between the rs1800896 polymorphism and the progression of leishmaniasis in the models: **(A)** dominant (GG+GA vs. AA), **(B)** recessive (GG vs. GA+AA), **(C)** allelic (G vs. A), **(D)** homozygous (GG vs. AA), and **(E)** heterozygous (GA vs. AA).

For the IL-10 SNP-1082, 4 studies were included in the final analysis [ 18, 30, 35, 36], including 341 cases and 369 controls. OR values showed no associations between disease development and genetic models: dominant (OR 1.02, 95% CI 0.70-1.50, I^2^ = 24%, p = 0.91), recessive (OR 0.74, 95% CI 0.36 -1.56, I^2^ = 57%, p = 0.43), allelic (OR 0.86, 95% CI 0.69-1.07, I^2^ = 0%, p = 0.18), homozygous (OR 0.98, 95% CI 0.56-1.72, I^2^ = 0%, p = 0.95), and heterozygous (OR 1.08, 95% CI 0.71-1.62, I^2^ = 38%, p = 0.72) ( Figure 5). Two control groups in this meta-analysis were not within HWE ( Table 2).

### Quality evaluation criteria

Scores ranged from 59% to 90% ( Table 3). Studies incomplete obtained scores between 59 to 68% [ 23, 24, 29, 32, 41]. Articles with slightly more detailed information obtained a percentage of around 77% [ 28, 30, 31, 33, 35, 38, 39]. Scores between 82 and 91% [ 14, 15, 16, 17, 18, 19, 20, 21, 22, 25, 26, 27, 34, 36, 37, 40] were given to those with great clarity. Therefore, the quality of the studies included in this review was considered satisfactory.

**Table 3.** Quality evaluation of the included studies.

Criteria	[14]	[15]	[16]	[17]	[18]	[19]	[20]	[21]	[22]	[23]	[24]	[25]	[26]
Question/objective sufficiently described?	2	2	2	2	2	2	2	2	2	2	2	2	2
Study design evident and appropriate?	2	2	2	2	2	2	2	2	2	2	2	2	2
Method of subject/comparison group selection or source of information/input variables described and appropriate?	1	2	2	2	2	2	2	2	2	2	1	2	2
Subject (and comparison group, if applicable) characteristics sufficiently described?	1	1	1	1	1	1	1	2	1	1	0	1	1
If interventional and random allocation was possible, was it described?	N/A	N/A	N/A	N/A	N/A	N/A	N/A	N/A	N/A	N/A	N/A	N/A	N/A
If interventional and blinding of investigators was possible, was it reported?	N/A	N/A	N/A	N/A	N/A	N/A	N/A	N/A	N/A	N/A	N/A	N/A	N/A
If interventional and blinding of subjects was possible, was it reported?	N/A	N/A	N/A	N/A	N/A	N/A	N/A	N/A	N/A	N/A	N/A	N/A	N/A
Criteria	[14]	[15]	[16]	[17]	[18]	[19]	[20]	[21]	[22]	[23]	[24]	[25]	[26]
Outcome and (if applicable) exposure measure(s) well defined and robust to measurement/misclassification bias? Means of assessment reported?	2	2	2	2	2	2	2	2	2	2	1	2	2
Sample size appropriate?	2	2	2	2	2	1	2	2	2	2	2	2	2
Analytic methods described/justified and appropriate?	2	2	2	2	2	2	2	2	2	1	2	2	2
Some estimate of variance is reported for the main results?	2	1	2	2	2	2	2	2	2	0	1	1	2
Controlled for confounding?	0	0	0	0	0	0	0	0	0	0	0	0	0
Results reported in sufficient detail?	2	2	2	2	2	2	2	2	2	1	1	2	2
Conclusions supported by the results?	2	2	2	2	2	2	2	2	2	1	1	2	2
**Maximum points**	22	22	22	22	22	22	22	22	22	22	22	22	22
**Total points**	18	18	19	19	19	18	19	20	19	14	13	18	19
**Summary score (%)**	82	82	86	89	86	82	86	91	86	64	59	82	86
Criteria		[27]	[28]	[29]	[30]	[31]	[32]	[33]	[34]	[35]	[36]	[37]	[38]
Question/objective sufficiently described?		2	2	2	2	2	1	1	2	2	2	2	2
Study design evident and appropriate?		2	2	2	2	2	2	2	2	2	2	2	2
Method of subject/comparison group selection or source of information/input variables described and appropriate?		2	2	1	1	2	1	2	2	2	2	2	2
Subject (and comparison group, if applicable) characteristics sufficiently described?		1	0	1	1	1	1	1	1	1	1	2	1
If interventional and random allocation was possible, was it described?		N/A	N/A	N/A	N/A	N/A	N/A	N/A	N/A	N/A	N/A	N/A	N/A
If interventional and blinding of investigators was possible, was it reported?		N/A	N/A	N/A	N/A	N/A	N/A	N/A	N/A	N/A	N/A	N/A	N/A
If interventional and blinding of subjects was possible, was it reported?		N/A	N/A	N/A	N/A	N/A	N/A	N/A	N/A	N/A	N/A	N/A	N/A
Outcome and (if applicable) exposure measure(s) well defined and robust to measurement/misclassification bias? Means of assessment reported?		2	2	2	2	2	2	2	2	2	2	2	2
Sample size appropriate?		2	1	2	1	2	1	2	2	1	2	2	2
Analytic methods described/justified and appropriate?		2	2	1	2	2	2	2	2	2	2	2	2
Criteria		[27]	[28]	[29]	[30]	[31]	[32]	[33]	[34]	[35]	[36]	[37]	[38]
Some estimate of variance is reported for the main results?		2	2	0	2	2	2	1	2	2	2	2	0
Controlled for confounding?		0	0	0	0	0	0 0	0	0	0	0	0 0	0
Results reported in sufficient detail?		2	2	1	2	2	2	2	2	2	2	2	2
Conclusions supported by the results?		2	2	1	2	2	2	2	2	2	2	2	2
**Maximum points**		22	22	22	22	22	22	22	22	22	22	22	22
**Total points**		19	17	13	17	17	15	17	19	17	19	20	17
**Summary score (%)**		86	77	59	77	77	68	77	86	77	86	91	77
Criteria					[39]			[40]			[41]		
Question/objective sufficiently described?					2			2					
Study design evident and appropriate?								2			2		
Method of subject/comparison group selection or source of information/input variables described and appropriate?					2			2			2		
Subject (and comparison group, if applicable) characteristics sufficiently described?					0			1			0		
If interventional and random allocation was possible, was it described?					N/A			N/A			N/A		
If interventional and blinding of investigators was possible, was it reported?					N/A			N/A			N/A		
If interventional and blinding of subjects was possible, was it reported?					N/A			N/A			N/A		
Outcome and (if applicable) exposure measure(s) well defined and robust to measurement/misclassification bias? Means of assessment reported?					2			2			1		
Sample size appropriate?					1			2			2		
Analytic methods described/justified and appropriate?					2			2			2		
Some estimate of variance is reported for the main results?					2			2			1		
Controlled for confounding?					0			0			0		
Results reported in sufficient detail?					2			2			1		
Conclusions supported by the results?					2			2			1		
**Maximum points**					22			22			22		
**Total points**					17			19			13		
**Summary score (%)**					77			86			59		

0 if the response is ‘no’; 1 if the response is ‘partial’; 2 if the response is ‘yes’ followed by N/A if not applicable.

## Discussion

VL is a complex multifactorial disease whose exact mechanisms leading to its development are not fully known. Factors such as host-parasite interactions, *Leishmania* species, environmental conditions, and immunological and genetic factors can contribute to the progression of the disease [ 3- 4]. Disease progression can be associated with the individual immunological profile. The resistance or susceptibility of clinical manifestations are associated with responses that are not yet fully understood, but single-base polymorphisms in immunological coding genes may be responsible for the change in this profile [ 3, 6, 42].

The association between the IFN-γ +874T/A polymorphism and VL progression has been studied in several studies [ 18, 29, 30], but they presented different results. Maghsood *et al*. [ 29] demonstrated that the rs2430561 polymorphism was not associated with the clinical features of the disease, but healthy seropositive individuals with the TT genotype showed greater secretion of this cytokine. Kalani *et al.* [ 18] concluded that the AT genotype is associated with susceptibility and that the TT genotype is associated with resistance in Iranians. However, Al-Bashier [ 30] concluded that the A allele was considered a risk factor for the development of the disease. Due to the importance of this cytokine in the immune responses associated with the development of VL and to better understand the role of this SNP, we performed this meta-analysis. Our results indicated that there was a significant association between rs2430561 and disease progression in the dominant model (OR 1.64, 95% CI 1.13-2.38; I^2^ = 0%, p = <0.01) and heterozygous model (OR 1.72, 95% CI 1.15-2.57; I^2^ = 0%, p = <0.01). These results indicate that the IFN-γ +874T/A polymorphism may play an important role in disease progression. According to the analysis of the dominant model, the +874 AA genotype was associated with a 1.64-fold increased risk of developing the disease. This result corroborates previous studies by Kalani *et al.* [ 18] and Al-Bashier [ 30]. This may be because the AA genotype leads to low production of IFN-γ, which may increase the chance of developing VL. However, these results must be interpreted with caution, as the low number of studies included in the analysis may have influenced the outcome.

Regarding rs187238 of the promoter region of the IL-18 gene, previous studies carried out with Indian and Iranian individuals did not show an association between this SNP and the progression of kala-azar [ 21, 22, 23]. In contrast, our recessive model demonstrated that the GG genotype was associated with a 1.33-fold increased risk of developing the disease. These differences in results may be associated with the limited number of patients and controls when carrying out an individual analysis of each study [ 22]. When carrying out a group analysis, these results may change due to the influence of the weight of all research. 

Previous studies between SNPs in the promoter region of the IL-10 gene have demonstrated an association between them and the development of VL. Hajilooi *et al.* [ 34], when evaluating rs1800871, demonstrated that the CT genotype was a risk factor for mortality in Iranians. In another study, Hajilooi *et al.* [ 36] demonstrated that the AG genotype of the region -1082 was also considered a risk factor for the disease. However, the results are contradictory, and other studies did not observe a significant association [ 18, 30, 35]. Our results indicate that there was no association between the presence of polymorphisms and resistance or susceptibility. This can be explained by the fact that vulnerability to VL may not be related only to these SNPs but also to several other characteristics, such as ethnicity, *Leishmania* species, and host conditions, among others, thus demonstrating that the mechanisms responsible for the disease they are caused by a combination of multigenetic and environmental factors [ 3, 34]. Furthermore, other components of innate and acquired immunity and other cytokines are also involved in the clinical progression of this disease. Besides, it may be related to the combined inheritance of SNP and polymorphic haplotypes that are inherited together by linkage blocks and may influence disease predisposition.

Finally, the limitations of the present study should be noted. In the final analyses, there are a few studies published and incorporated into the meta-analysis for all SNPs, which may have somehow altered the results. Furthermore, there were methodological differences between the studies, such as variation in the types of control groups, ethnicity, lack of a single standard test for detecting VL, and others. These characteristics may have affected the analyses, the forest plot, and the heterogeneity values.

In conclusion, the main clinical outcomes found were: susceptibility -511CC genotype of IL-1β, rs2227513 (AG genotype) of IL-22 in Iranians. Resistance: -511 TT of IL-1β, T allele of the region -656G/T, CC genotype at position -607 of IL-18, TT genotype of +874 A/T of IFN-γ in Iranians. Risk factor: TT genotype in the -251 T/A of IL-8, AG genotype of the region -1082 (rs1800896) in Iranians, T allele of region -509 of TGF-β1 in Brazilians. Protective factor: G allele of the region -656G/T of IL-18 in Indians, A allele, and the AA genotype of rs1026786 of IL-22 in Iranians.

In addition, this meta-analysis demonstrated that the +874 AA polymorphisms of IFN-γ and -137GG of the IL-18 gene may be associated with the risk of developing VL; however, these data should be interpreted with caution due to the small sample size. Therefore, future research on this topic with greater methodological rigor is necessary to better understand the relationship between SNPs and the progression of VL. Understanding molecular conditions may aid in the discovery of new therapeutic targets for future pharmacological interventions.

### Abbreviations

ARMS-PCR: amplification refractory mutation system-polymerase chain reaction; AS-PCR: allele-specific polymerase chain reaction; IL: interleukin; PCR-RFLP: polymerase chain reaction-restriction fragment length polymorphism; SNP: single nucleotide polymorphism: *Th1*: T helper 1; *Th17*: T helper 17; *Th2*: T helper 2; VL: visceral leishmaniasis; WHO: World Health Organization.

## Data Availability

All data generated or analyzed during this study are included in this article.

## Supplementary material

The following online material is available for this article:

Additional file 1. Registration of the systematic review protocol in the Prospective International Registry Platform for Systematic Reviews (PROSPERO).

Additional file 2. Search strategy used in each database.
