# Supplementary material for: Human visceral leishmaniasis and polymorphisms in interleukin-coding genes: a systematic review
Source: J Venom Anim Toxins Incl Trop Dis. 2024 Oct 18;30:e20240018. doi: 10.1590/1678-9199-JVATITD-2024-0018 (PMC11493340; doi:10.1590/1678-9199-JVATITD-2024-0018)
Supplement: Additional file 1. [file 1678-9199-jvatitd-30-e20240018-s1.pdf]

J Venom Anim Toxins incl Trop Dis
https://doi.org/10.1590/1678-9199-JVATITD-2024-0018

Supplementary Material to "Human visceral leishmaniasis and polymorphisms in interleukin-coding genes: a systematic review"

Additional file 1. Registration of the systematic review protocol in the Prospective International Registry Platform for Systematic Reviews (PROSPERO).

NIHR | National Institute for Health Research

PROSPERO
International prospective register of systematic reviews

Gene polymorphisms and human visceral leishmaniasis.

To enable PROSPERO to focus on COVID-19 submissions, this registration record has undergone basic automated checks for eligibility and is published exactly as submitted. PROSPERO has never provided peer review, and usual checking by the PROSPERO team does not endorse content. Therefore, automatically published records should be treated as any other PROSPERO registration. Further detail is provided here.

Citation

Amanda Vieira, Manuela Menezes, Pablo Cantalice, Elis Dionísio, Walter Barbosa, Zulma Medeiros. Gene polymorphisms and human visceral leishmaniasis.. PROSPERO 2022 CRD42022350889 Available from: https://www.crd.york.ac.uk/prospero/display_record.php?ID=CRD42022350889

Review question

Can genetic polymorphisms in human visceral leishmaniasis affect susceptibility and/or resistance to the development of the disease?

Searches

Included databases will be: EMBASE, Web of Science, PubMed, Scopus and LILACS. Databases will be searched without time restriction.

Types of study to be included

All types of studies that evaluated polymorphisms associated with susceptibility and/or resistance to visceral leishmaniasis in humans [except systematic review (with or without meta-analysis), randomized studies].

Condition or domain being studied

Visceral Leishmaniasis (VL) is a neglected disease that affects tropical and subtropical regions, with high mortality rates when early treatment is not instituted. Approximately 50, 000 to 90, 000 cases occur annually, with more than 90% concentrated in 10 countries around the world: Brazil, China, Ethiopia, India, Iraq, Kenya, Nepal, Somalia, South Sudan and Sudan. The development of the disease is triggered by several factors such as: age, presence of associated comorbidities, nutrition, immune and genetic characteristics. The presence of gene polymorphisms can trigger processes of susceptibility or protection to the disease. Therefore, the understanding of the action of these polymorphisms becomes necessary for the improvement in the treatment and directed to the patient. As well, it may contribute to the identification of new immunogenetic biomarkers.

Participants/population

Studies that analyzed gene polymorphisms related to the development of human visceral leishmaniasis.

Intervention(s), exposure(s)

None.

Comparator(s)/control

None.

Main outcome(s)

Resistant and/or susceptible individuals to the development of visceral leishmaniasis.

Measures of effect

Measures of p value for observation of resistance and susceptibility profiles.

Odds ratios (OR).

Additional outcome(s)

None.

Data extraction (selection and coding)

The references will be screened against predetermined eligibility criteria for inclusion in the review.

We will extract data including author, year, country, polymorphism analysis, outcome, sample size, genotyping method.

Initially, duplicate jobs will be deleted. After this stage, the studies will be sorted through titles and abstracts (first phase). Those selected will undergo a complete reading and extraction of the data. All selection steps will be done by two independent reviewers, if there is any disagreement, a third reviewer (with more experience) will be contacted.

All phases of the screening were supported by the application: Rayyan (Ouzzani, Mourad, et al. "Rayyan—a web and mobile app for systematic reviews." Systematic reviews 5.1 (2016): 1-10).

Risk of bias (quality) assessment

The Risk of bias assessment will be performed by two graduate students (Amanda Vieira and Manuela Menezes) and any disagreement will be resolved by a third person (Zulma Maria de Medeiros) who has experience in visceral leishmaniasis research. Quality of studies will be evaluated by Standard Quality Assessment Criteria for Evaluation of Primary Research Papers from a Variety of Fields (Kmet LM, Lee RC, Cook LS Standard quality assessment criteria for evaluating primary research papers from a variety of fields. Edmonton: Alberta Heritage Foundation for Medical Research; 2004, 1–22).

Strategy for data synthesis

The ORs and 95% CIs will be measured. Differences will be considered significant for p values < 0.05. The magnitude of these associations will be estimated as OR, using 95% confidence intervals.

Analysis of subgroups or subsets

The alleles present in each polymorphism, analyzed from each genetic model, will be evaluated at the subgroup level. With the purpose of possible association between the frame of resistance and/or susceptibility related to VL and the genetic alteration.

Contact details for further information

Amanda Vieira

amandaa-vieira@outlook.com

Organisational affiliation of the review

UPE - Universidade de Pernambuco.

Review team members and their organisational affiliations [1 change]

Miss Amanda Vieira. Universidade de Pernambuco - UPE.
Miss Manuela Menezes. Instituto Aggeu Magalhães, FIOCRUZ-PE.
Mr Pablo Cantalice. Universidade Federal de Pernambuco - UFPE
Miss Elis Dionísio. Universidade Federal do Amazonas - UFAM
Mr Walter Barbosa. Instituto Aggeu Magalhães, FIOCRUZ - PE
Professor Zulma Medeiros. Universidade de Pernambuco - UPE

Type and method of review

Diagnostic, Systematic review

Anticipated or actual start date

31 October 2022

Anticipated completion date [1 change]

31 October 2024

Funding sources/sponsors

None.

Conflicts of interest

Language

English

Country

Brazil

Published protocol

https://www.crd.york.ac.uk/prospero/display_record.php?ID=CRD42022350889

Stage of review [2 changes]

Review Ongoing

Subject index terms status

Subject indexing assigned by CRD

Subject index terms

Humans; Leishmaniasis, Visceral; Polymorphism, Genetic

Date of registration in PROSPERO

13 August 2022

Date of first submission

02 August 2022

Stage of review at time of this submission [2 changes]

Stage	Started	Completed
Preliminary searches	Yes	Yes
Piloting of the study selection process	Yes	Yes
Formal screening of search results against eligibility criteria	Yes	Yes
Data extraction	Yes	Yes
Risk of bias (quality) assessment	Yes	Yes
Data analysis	Yes	Yes

Revision note

This review protocol was changed to update important information, with the progress of searches, analysis, publication forecast, among others.

The record owner confirms that the information they have supplied for this submission is accurate and complete and they understand that deliberate provision of inaccurate information or omission of data may be construed as scientific misconduct.

The record owner confirms that they will update the status of the review when it is completed and will add publication details in due course.

Versions

13 August 2022

13 August 2022

15 August 2022

03 July 2024

03 July 2024
