# Supplementary material for: Human visceral leishmaniasis and polymorphisms in interleukin-coding genes: a systematic review
Source: J Venom Anim Toxins Incl Trop Dis. 2024 Oct 18;30:e20240018. doi: 10.1590/1678-9199-JVATITD-2024-0018 (PMC11493340; doi:10.1590/1678-9199-JVATITD-2024-0018)
Supplement: Additional file 2. [file 1678-9199-jvatitd-30-e20240018-s2.pdf]

J Venom Anim Toxins incl Trop Dis
https://doi.org/10.1590/1678-9199-JVATITD-2024-0018

Supplementary Material to "Human visceral leishmaniasis and polymorphisms in interleukin-coding genes: a systematic review"

Additional file 2. Search strategy used in each database.

PubMed

("visceral Leishmaniasis" OR "Black Fever" OR "Fever-Black" OR "Kala-Azar" OR "Kala Azar" OR **"Leishmaniasis, Visceral"[Mesh]**) AND ("Gene Polymorphism" OR "Gene Polymorphisms" OR "Polymorphism, Gene" OR "Polymorphisms, Gene" OR "Genetic Polymorphism" OR "Genetic Polymorphisms" OR "Polymorphism (Genetic)" OR "Polymorphisms (Genetics)" OR "Polymorphisms, Genetic" OR "**Polymorphism, Genetic"[MESH]**) AND (human OR "**Humans"[Mesh]**)

PubMed

#1 "visceral Leishmaniasis" (All Fields) (13.577) # 2

"Black Fever" (All Fields) (19)

3 "Fever-Black" (All Fields) (3)

4 "Kala-Azar" (All Fields) (3.057) # 5

"Kala Azar" (All Fields) (3.057)

6 "Leishmaniasis, Visceral"**[Mesh]** (All Fields) (11.056) #1 or

#2 or #3 or #4 or #5 or #6 (All Fields) (14.143)

#8 "Gene Polymorphism" (All Fields) (16.925)

#9 "Gene Polymorphisms" (All Fields) (22.596)

#10 "Polymorphism, Gene" (All Fields) (132)

#11 “Polymorphisms, Gene” (All Fields) (81)

#12 “Genetic Polymorphism” (All Fields) (126.321)

#13 “Genetic Polymorphisms” (All Fields) (19.307)

#14 “Polymorphism (Genetic)” (All Fields) (120.650)

#15 “Polymorphisms (Genetics)” (All Fields) (4)

#16 “Polymorphisms, Genetic” (All Fields) (67)

#17 “Polymorphism, Genetic”**[MESH]** (All Fields) (303.535)

#8 or #9 or #10 or #11 or #12 or #13 or #14 or #15 or #16 or #17 (All Fields) (318.764)

#18 human (All Fields) (22.279.690)

#19 **"Humans"[Mesh]** (All Fields) (21.409.210)

#19 or #20 (All Fields) (22.279.690)

((#1 or #2 or #3 or #4 or #5 or #6) AND (#8 or #9 or #10 or #11 or #12 or #13 or #14 or #15 or #16 or #17)) AND (#19 or #20) (All Fields) (195)

EMBASE

('visceral leishmaniasis'/exp OR 'visceral leishmaniasis' OR 'black fever'/exp OR 'black fever' OR 'fever-black' OR 'kala-azar'/exp OR 'kala-azar' OR 'kala azar'/exp OR 'kala azar' OR 'leishmaniasis, visceral'/exp OR 'leishmaniasis, visceral') AND ('gene polymorphism'/exp OR 'gene polymorphism' OR 'gene polymorphisms' OR 'polymorphism, gene' OR 'polymorphisms, gene' OR 'genetic polymorphism'/exp OR 'genetic polymorphism' OR 'genetic polymorphisms' OR 'polymorphism (genetic)'/exp OR 'polymorphism (genetic)' OR 'polymorphisms (genetics)' OR 'polymorphisms, genetic' OR 'polymorphism, genetic'/exp OR 'polymorphism, genetic') AND ('human'/exp OR human OR 'humans'/exp OR 'humans')

#1 ('visceral leishmaniasis'/exp OR 'visceral leishmaniasis' OR 'black fever'/exp OR 'black fever' OR 'fever-black' OR 'kala-azar'/exp OR 'kala-azar' OR 'kala azar'/exp OR 'kala azar' OR 'leishmaniasis, visceral'/exp OR 'leishmaniasis, visceral') (All Fields) (15.528)

#2 ('gene polymorphism'/exp OR 'gene polymorphism' OR 'gene polymorphisms' OR 'polymorphism, gene' OR 'polymorphisms, gene' OR 'genetic polymorphism'/exp OR 'genetic polymorphism' OR 'genetic polymorphisms' OR 'polymorphism (genetic)'/exp OR 'polymorphism (genetic)' OR 'polymorphisms (genetics)' OR 'polymorphisms, genetic' OR 'polymorphism, genetic'/exp OR 'polymorphism, genetic') (All Fields) (494.768)

#3 ('human'/exp OR human OR 'humans'/exp OR 'humans') (All Fields) (27.748.126) #1 AND #2 AND #3 (All Fields) (199)

SCOPUS

(TITLE-ABS-KEY (("visceral Leishmaniasis" OR "Black Fever" OR "Fever-Black" OR "Kala-Azar" OR "Kala Azar" OR "Leishmaniasis, Visceral")) AND TITLE-ABS-KEY (("Gene Polymorphism" OR "Gene Polymorphisms" OR "Polymorphism, Gene" OR "Polymorphisms, Gene" OR "Genetic Polymorphism" OR "Genetic Polymorphisms" OR "Polymorphism (Genetic)" OR "Polymorphisms (Genetics)" OR "Polymorphisms, Genetic" OR "Polymorphism, Genetic")) TITLE-ABS-KEY AND ((human OR "Humans")))

#1 ("visceral Leishmaniasis" OR "Black Fever" OR "Fever-Black" OR "Kala-Azar" OR "Kala Azar" OR "Leishmaniasis, Visceral") (Article title, Abstract, Keywords) (16.526)

#2 ("Gene Polymorphism" OR "Gene Polymorphisms" OR "Polymorphism, Gene" OR "Polymorphisms, Gene" OR "Genetic Polymorphism" OR "Genetic Polymorphisms" OR "Polymorphism (Genetic)" OR "Polymorphisms (Genetics)" OR "Polymorphisms, Genetic" OR "Polymorphism, Genetic") (Article title, Abstract, Keywords) (206.110)

#3 (human OR "Humans") (Article title, Abstract, Keywords) (26.084.444) #1 AND
#2 AND #3 (Article title, Abstract, Keywords) (111)

Web of Science

(“visceral Leishmaniasis" OR "Black Fever" OR "Fever-Black" OR "Kala-Azar" OR "Kala Azar" OR "Leishmaniasis, Visceral") AND ("Gene Polymorphism" OR "Gene Polymorphisms" OR "Polymorphism, Gene" OR "Polymorphisms, Gene" OR "Genetic Polymorphism" OR "Genetic Polymorphisms" OR "Polymorphism (Genetic)" OR "Polymorphisms (Genetics)" OR "Polymorphisms, Genetic" OR "Polymorphism, Genetic") (human OR "Humans")

#1 ("visceral Leishmaniasis" OR "Black Fever" OR "Fever-Black" OR "Kala-Azar" OR "Kala Azar" OR "Leishmaniasis, Visceral") (All Fields) (14.865)

#2 ("Gene Polymorphism" OR "Gene Polymorphisms" OR "Polymorphism, Gene" OR "Polymorphisms, Gene" OR "Genetic Polymorphism" OR "Genetic Polymorphisms" OR "Polymorphism (Genetic)" OR "Polymorphisms (Genetics)" OR "Polymorphisms, Genetic" OR "Polymorphism, Genetic") (All Fields) (91.509)

#3 (human OR "Humans") (All Fields) (8.046.244)

#1 AND #2 AND #3 (All Fields) (22)

SciELO

(*"Leishmaniose Visceral") OR (*"Calazar") AND ("Polimorfismo Genético") OR ("Polimorfismo (Genético)") OR ("Polimorfismo de Nucleotídeo Único") OR ("Polimorfismo de Um Único Nucleotídeo") OR ("SNPs")

#1 (*"Leishmaniose Visceral") OR (*"Calazar")

#2 AND ("Polimorfismo Genético") OR ("Polimorfismo (Genética)") OR ("Polimorfismo de Nucleotídeo Único") OR ("Polimorfismo de Um Único Nucleotídeo") OR ("SNPs")

#1 AND #2 (All Fields) (61)
